# Tuberculosis of the Cervical Vertebrae With Retropharyngeal and Parapharyngeal Abscesses Due to Staphylococcus aureus and Mycobacterium tuberculosis in an Adult: A Report of a Rare Case

**DOI:** 10.7759/cureus.61412

**Published:** 2024-05-31

**Authors:** Sankalp Yadav

**Affiliations:** 1 Medicine, Shri Madan Lal Khurana Chest Clinic, New Delhi, IND

**Keywords:** cartridge-based nucleic acid amplification test, parapharyngeal abscesses, retropharyngeal abscess, tuberculosis, pott's disease

## Abstract

Tuberculosis is a disease with presentations both in the lungs and at other extrapulmonary sites. While pulmonary tuberculosis constitutes a significant proportion of total tuberculosis cases, extrapulmonary cases with infections at rare sites are also documented. Herein, an exceedingly rare case of tuberculosis of the cervical vertebrae with retropharyngeal and parapharyngeal abscesses due to *Staphylococcus aureus* and *Mycobacterium tuberculosis* in a young Indian male is presented. The rarity of the locations of the lesions with coinfections with two bacteria made the diagnosis challenging. Besides, the potential for a retropharyngeal abscess to compress the airway is an emergency situation. However, the ultimate diagnosis was achieved with the help of a radiograph of the neck, contrast-enhanced computed tomography of the neck, fine-needle aspiration cytology, and a cartridge-based nucleic acid amplification test. He was initiated on appropriate antibiotics and antituberculous chemotherapy per his weight.

## Introduction

Tuberculosis of the spine is known as Pott's disease [1]. It is mostly seen in the thoraco-lumbar region, followed by the cervical vertebrae [2]. Oftentimes, the infection of the cervical vertebrae by *Mycobacterium tuberculosis *results in the accumulation of pus in the retropharyngeal and parapharyngeal regions [3]. This is an emergency situation due to the potential for these abscesses to compress the airway [3,4]. It is usually seen in children below five years of age and is exceedingly rare after this age [3]. Additionally, the classical features of tuberculosis are mostly missing, which makes the diagnosis an arduous task [2].

Further, a diagnostic delay could result in fatal outcomes due to other complications like mediastinitis, jugular necrotizing fasciitis, aspiration pneumonia, and empyema [5]. A case of tuberculosis of the cervical vertebrae with retropharyngeal and parapharyngeal abscesses due to *Staphylococcus aureus *and *Mycobacterium tuberculosis* in an 18-year-old Indian male is presented, who was successfully diagnosed with a high degree of clinical suspicion.

## Case presentation

An 18-year-old non-diabetic Indian male from a low socioeconomic background presented with chief complaints of upper backache for two months, followed by right-sided neck swelling for one and a half months. He also complained of voice changes and difficulty swallowing for 15 days. Dysphagia was initially associated with solid food, progressing to liquids as well. Additionally, he complained of difficulty breathing in the supine position, snoring, and drooling saliva in the morning for 15 days. He also reported developing right-sided torticollis one month ago, which was partially resolved after taking physiotherapy at a local center.

There was no history of fever, weight loss, night sweats, or any other constitutional symptoms of tuberculosis. He had a history of trauma to the neck on the right side when he fell from the three-wheeler public transport. Further, there was no history of similar complaints or tuberculosis in the family or any of his acquaintances. And there was no history of dental pain, recurrent sore throat, or neck instrumentation.

A general examination was suggestive of a young male with a mesomorphic build. He was hemodynamically stable, and there was no pallor, cyanosis, clubbing, icterus, or edema. Moreover, his systemic examination was unremarkable.

Local examination of the neck revealed a 2 cm × 2 cm soft, fluctuant diffuse swelling in the right posterior triangle at level II. There were no discharging sinuses or erythema. The movement of the neck was painful, with restrictive torticollis on the right side. There was notable kyphosis at the C4-C5 level. Throat examination was suggestive of reduced mouth opening with the presence of sludge on the posterior pharyngeal wall (right > left). Mild stridor was present. Indirect laryngoscopy could not be done due to a midline bulge in the posterior pharyngeal wall.

**Figure 1:**
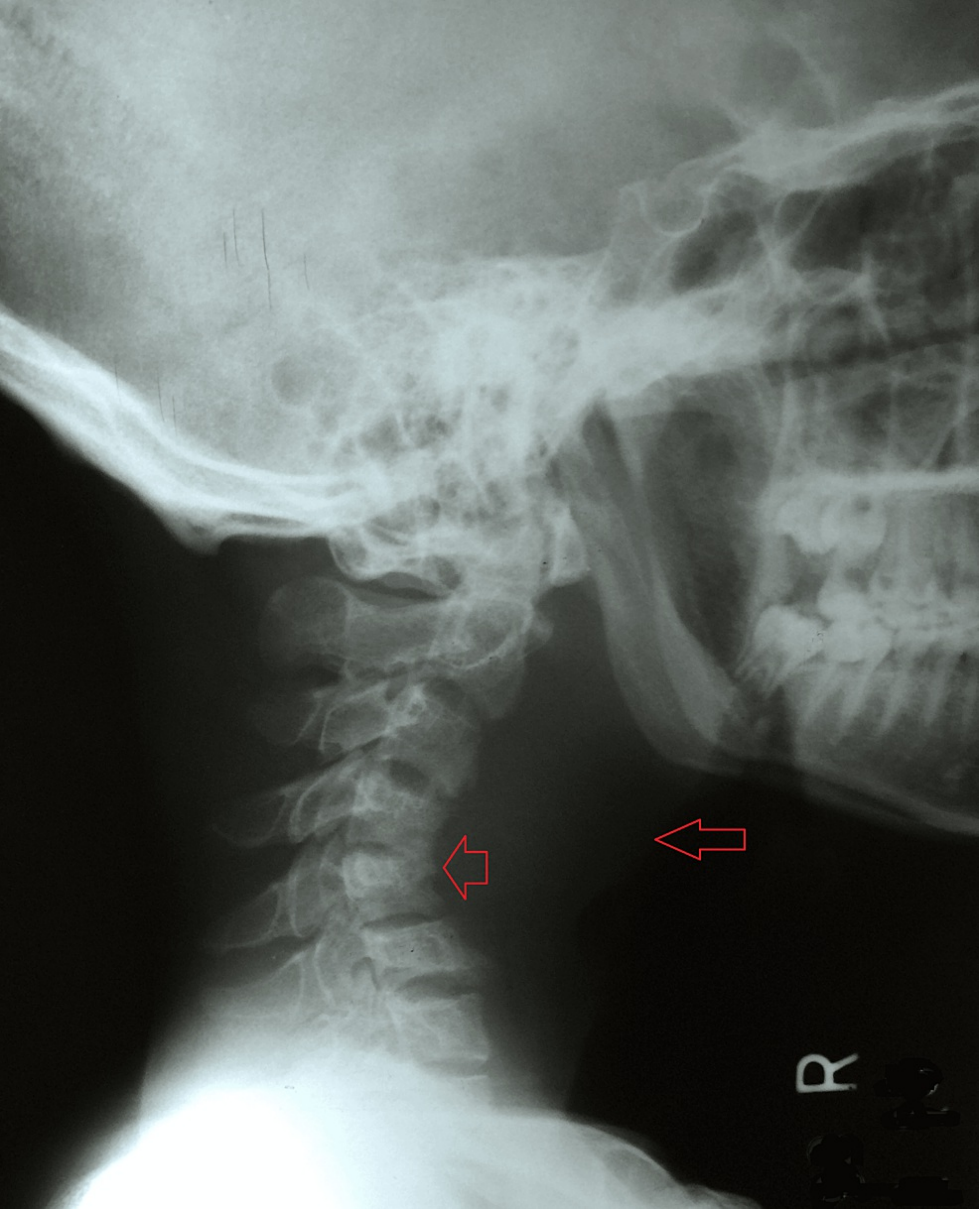
A plain radiograph of the neck suggestive of increased prevertebral space with reduced joint space between C4-C5 and C6-C7 vertebrae, loss of normal cervical lordosis, and collapse of the C4 vertebra.

A plain radiograph of the neck was suggestive of increased prevertebral space with reduced joint space between C4-C5 and C6-C7 vertebrae, loss of normal cervical lordosis, and collapse of the C4 vertebra (Figure 1).

Routine blood investigations were remarkable for a hemoglobin of 11.9 g/dl and an erythrocyte sedimentation rate of 76 mm in the first hour. The rest of the blood panel, including HIV and hepatitis, was unremarkable. Fine-needle aspiration of the right cervical swelling was done at a different center, and 3 ml of purulent material was aspirated. Histopathology was suggestive of mainly necrotic bodies with degenerating polymorphs, lymphocytes, and a few Langhans giant cells. Ziehl-Neelsen staining was negative for acid-fast bacilli. An XpertGene/cartridge-based nucleic acid amplification test was not suggestive of *Mycobacterium tuberculosis*. An aspiration of the pus from the posterior pharyngeal wall was done at the ear, nose, and throat minor operation theatre, and 34 ml of yellow-colored, blood-tinged pus was aspirated. A cartridge-based nucleic acid amplification test was repeated on the pus sample, and the results were suggestive of low detection of *Mycobacterium tuberculosis*. A culture was suggestive of coagulase-negative *Staphylococcus aureus* with resistance to penicillin on the 40th day. Further, the patient developed pus on multiple occasions, and the same was aspirated, as detailed in Table 1.

**Table 1:** The amount of pus aspirated and the duration from the time of presentation.

Episodes	The amount of pus aspirated
First (at the presentation)	34 ml
Second (10th day)	30 ml
Third (20th day)	17 ml
Fourth (31st day)	5 ml

**Figure 2:**
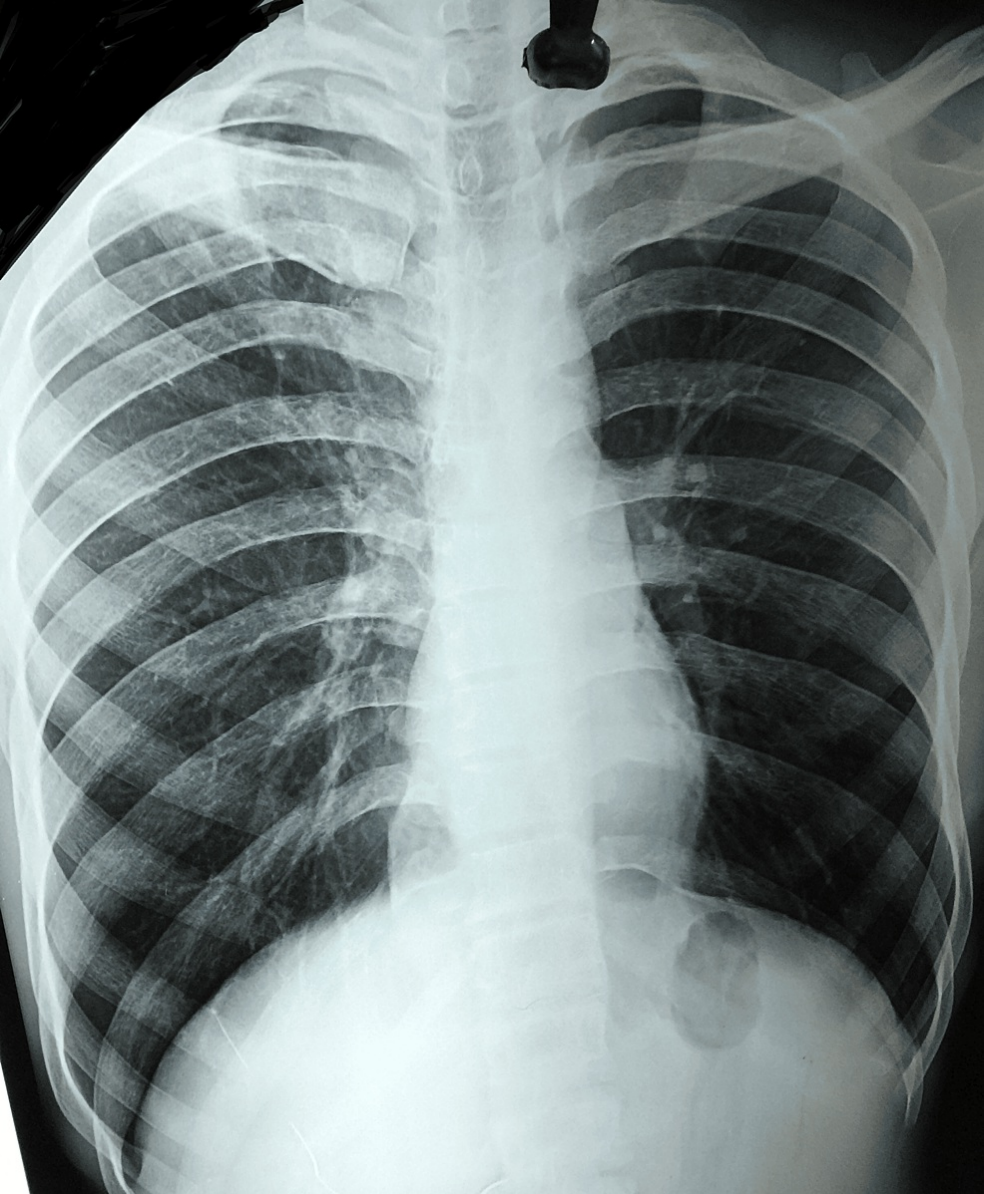
A normal chest radiograph.

A broad-spectrum antibiotic (Amoxyclav 625 mg) was initiated until the results of the culture were available. The patient's chest radiograph was normal (Figure 2).

**Figure 3:**
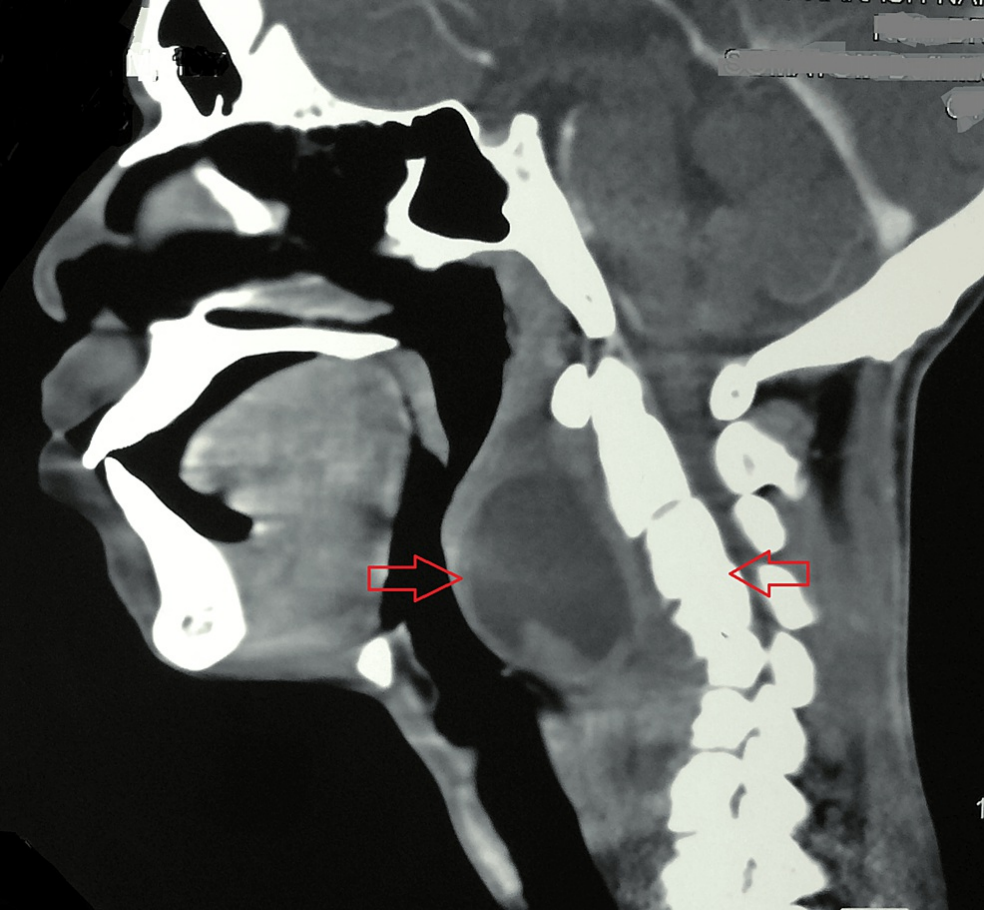
A CECT of the neck suggestive of a reversal of cervical lordosis with destruction of the C5 vertebral body and focal erosion of the adjacent C4 vertebral body with reduced intervertebral disc space at these levels. An associated hypodense collection, with enhancing rim and septae within, was seen in the anterior prevertebral region, with extension into retropharyngeal and parapharyngeal space in the midline extending from the level of the occipital condyle to the C7 vertebrae. CECT: contrast-enhanced computed tomography

**Figure 4:**
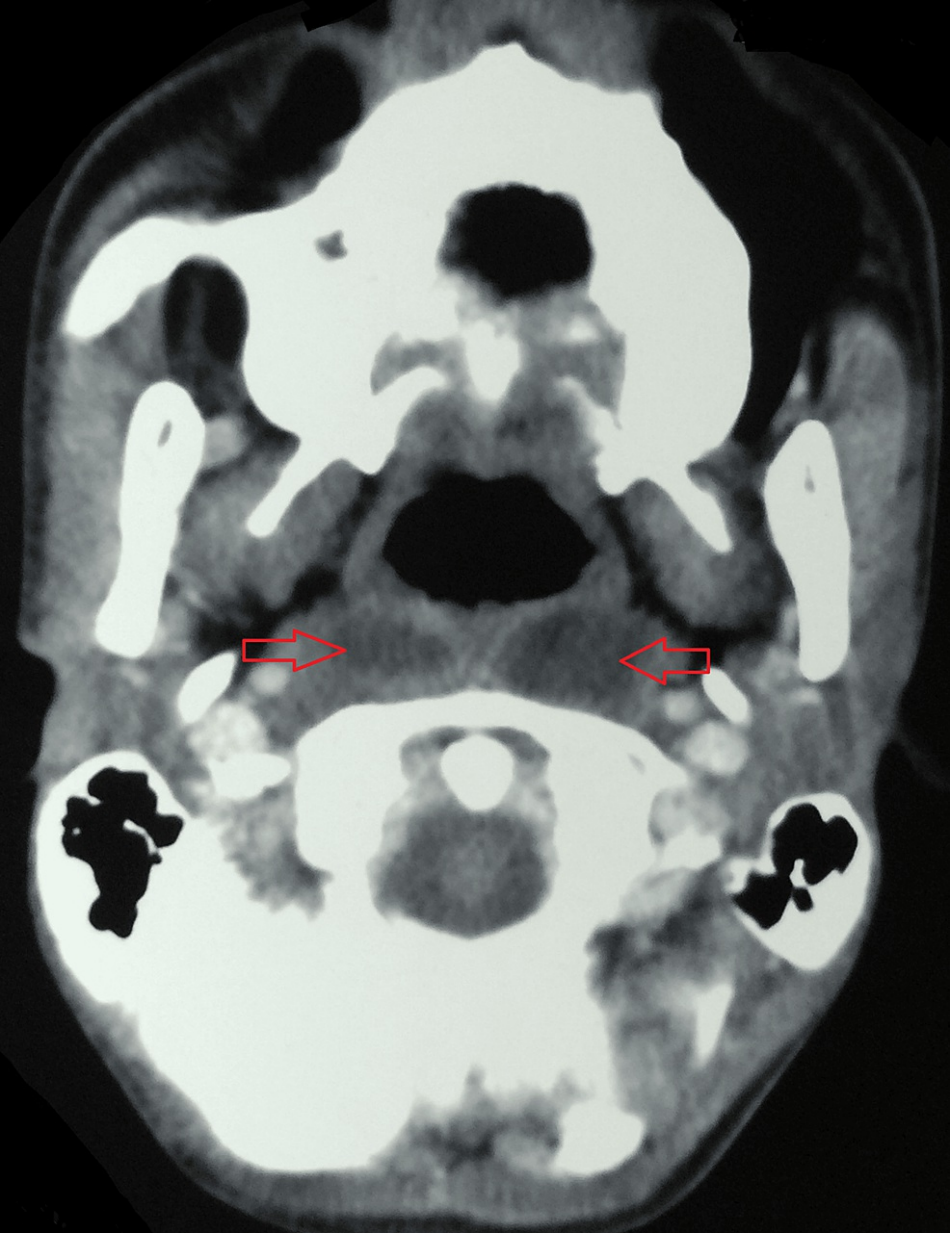
A CECT of the neck (axial view) suggestive of retropharyngeal and parapharyngeal abscess space in the midline extending. CECT: contrast-enhanced computed tomography

A contrast-enhanced computed tomography (CECT) of the neck was suggestive of a reversal of cervical lordosis. There was evidence of erosion with destruction of the C5 vertebral body and focal erosion of the adjacent C4 vertebral body with reduced intervertebral disc space at these levels. An associated hypodense collection, with enhancing rim and septae within, was seen in the anterior prevertebral region, with extension into retropharyngeal and parapharyngeal space in the midline extending from the level of the occipital condyle to the C7 vertebrae. Laterally, it was extending to the carotid space bilaterally (Figure 3 and Figure 4).

**Figure 5:**
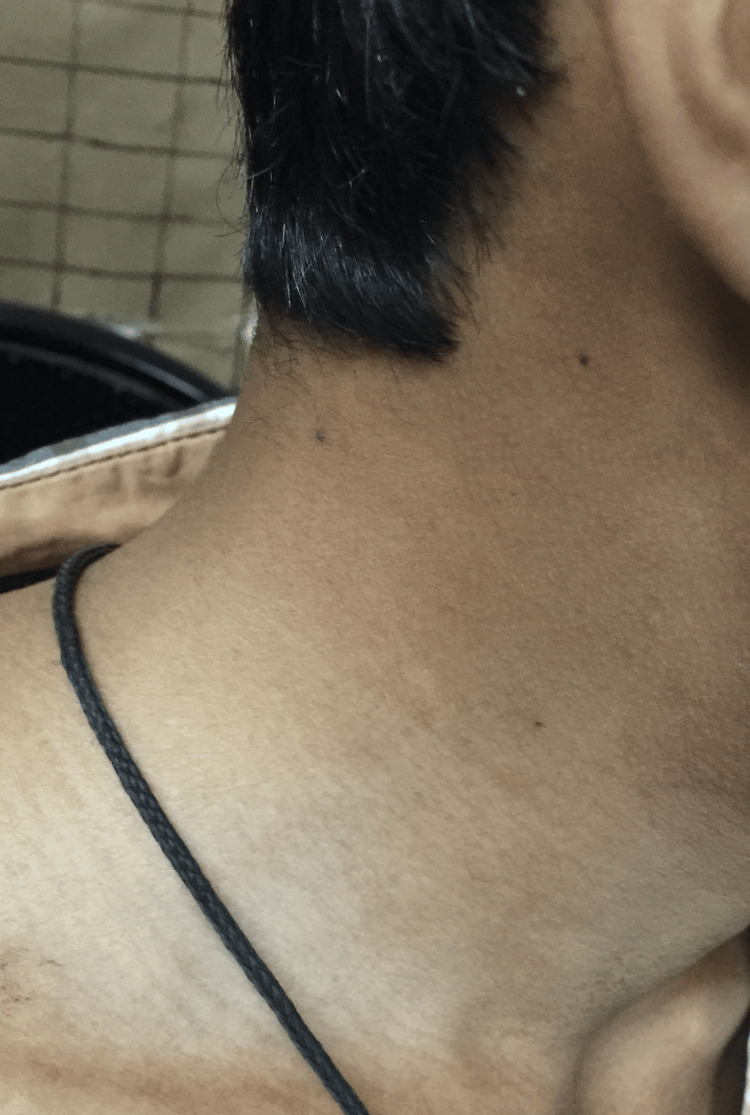
Gross image at the third-month follow-up showing no clinical deterioration.

A final diagnosis of cervical Pott's spine of the C3, C4, C5, and C6 without distal neurovascular deficit with retropharyngeal and parapharyngeal abscess due to* Staphylococcus aureus *and *Mycobacterium tuberculosis *was made, and the patient was initiated on antituberculous therapy with four drugs, i.e., rifampicin, ethambutol, pyrazinamide, and isoniazid, for 56 days, followed by a 10-month continuation phase with three antituberculous drugs, i.e., isoniazid, rifampicin, and ethambutol, in fixed drug combinations of antituberculous drugs. Additionally, tablet clindamycin (600 mg) was added twice daily for seven days. He was also prescribed a combination of paracetamol, ibuprofen, and chlorzoxazone twice daily, tablet ranitidine 150 mg twice daily, and Betadine gargles three times daily. A Philadelphia collar was advised. He was counseled for treatment adherence and regular follow-ups in the orthopedic, ear, nose, and throat (ENT), and infectious disease outpatient departments. He fared well on the treatment, with an evident improvement in the condition with the disappearance of the right-sided swelling, marked improvement in dysphagia and dyspnea, and reduction of pain in neck movement (Figure 5).

The patient was followed up for three months, but he requested a transfer to his village, which was granted. A last follow-up at 12 was done in his village, but the reports were inaccessible. However, his outcome was mentioned as cured at 12 months in the national tuberculosis portal, i.e., Nikshay [6].

## Discussion

*Mycobacterium tuberculosis* infection of the cervical spine is exceedingly rare and reported in nearly 0.03% of cases [7]. Pott's disease of the cervical spine, resulting in the destruction and erosion of the vertebrae, can result in prevertebral collections like retropharyngeal and parapharyngeal abscesses [8]. Retropharyngeal abscess is commonly of bacterial origin and is rarely an entity. Unusual in adults, it could be due to trauma to the posterior mucosal wall of the pharynx and cervical part of the oesophagus by a sharp foreign body [9]. Moreover, it is frequently observed in patients with impaired immune systems, and endoscopic operations and endotracheal intubation are typically associated with it [3].

Diagnosis of retropharyngeal abscesses is challenging due to the ambiguity of the clinical features with other infections [2]. *Streptococcus viridans* was the most frequently involved pathogen in a systemic review of 210 cases conducted by Parhiscar and Har-El, followed by *Staphylococcus epidermidis *(22%) and *Staphylococcus aureus* (22%) [10]. In contrast, *Streptococcus pyogenes *was the most frequently isolated pathogen in a clinical review conducted by Goldenberg et al. [11]. Retropharyngeal abscess is an uncommon tuberculosis presentation that can be caused by either tubercular involvement of the cervical spine or, more frequently, tubercular involvement of the lymph nodes in the retropharyngeal space [12].

Sore throat, fever, dysphagia, odynophagia, painful neck motions, and breathing difficulties are among the main symptoms [13]. Upon examination, stridor, saliva drooling, palpable neck mass, posterior pharyngeal wall bulging, and neck muscle spasm may be noted. Radiographs of the lateral cervical spine may show soft tissue enlargement, air pockets, osteolytic lesions in the vertebrae, and loss of lordosis in the cervical spine [14]. Advanced radiometric investigations are helpful in determining the extent of the involvement and precise locations of the lesions and pus [3].

Management involves drainage of the pus followed by antituberculous therapy [2]. For Pott's spine, a 12-month treatment with antituberculous drugs is indicated. Afterwards, a decision to extend the treatment is based on a clinical assessment [15].

Due to consequences such as airway obstruction, aspiration pneumonia, epidural abscess, erosion into the carotid artery, sepsis, and jugular vein thrombosis, delayed treatment is linked with fatal outcomes [4].

A case of retropharyngeal and parapharyngeal abscesses due to the coinfection of *Staphylococcus aureus* and *Mycobacterium tuberculosis* was reported in a five-week-old boy by Shin et al. [16]. However, the present case differs from theirs in the presence of a cervical Pott's spine, ethnicity, and age. Also, the present case is an exceedingly rare case with simultaneous involvement of the tuberculosis of the cervical vertebrae with retropharyngeal and parapharyngeal abscesses due to *Staphylococcus aureus* and *Mycobacterium tuberculosis *in an adult male, which is not documented in the literature.

## Conclusions

A case of tuberculosis of the cervical vertebrae with retropharyngeal and parapharyngeal abscesses due to *Staphylococcus aureus *and *Mycobacterium tuberculosis* in a young Indian male is presented. The case emphasizes the need for comprehensive diagnostic work backed by a high degree of clinical suspicion to determine a final diagnosis. This case also stresses the need for the dissemination of information about such rare presentations, as even in endemic settings, there is a paucity of information about simultaneous infections of the cervical spine with retropharyngeal and parapharyngeal abscesses.
